# Cortico‐hippocampal dysfunction in APP‐KI and PS1 mouse models

**DOI:** 10.1002/alz.087004

**Published:** 2025-01-03

**Authors:** Melissa Hernández‐Frausto, Francesca Chinea, Jayeeta Basu

**Affiliations:** ^1^ Neuroscience Institute, NYU Langone Health, NYU Grossman School of Medicine, New York, NY USA; ^2^ Harvard University, Department of Neurology, Massachusetts General Hospital, Boston, MA USA

## Abstract

**Background:**

Lateral entorhinal cortex (LEC), medial entorhinal cortex (MEC), and hippocampal area exhibit cell damage due to B‐Amyloid depositions, intracellular tau aggregates, and neurofibrillay tangles. AD mouse models allow us to investigate how AD neurodegeneration affects specific brains circuits and resulting behaviors. Specifically, the PS1 and APP NL‐G‐F knock in (APP‐KI) mouse models are relevant due to their genetic modifications, episodic memory impairment, early AD pathophysiology, and novelty designed for study. Nevertheless, the circuit mapping of entorhinal cortex in the medial and lateral portion within the hippocampal area during the progression of AD remains elusive.

**Method:**

By using anatomical tracing and cognitive freely moving behaviors we compared the progression of cognitive deficits in the PS1 and APP‐KI mouse models. First, with AAV viral infection we expressed a green fluorophore in excitatory cell bodies in LEC and MEC, we examined through confocal microscopy the density of the projections in hippocampal area. Then, we compared with the behavioral performance in two cognitive tasks involved in the cortico‐hippocampal function; Novel Object Recognition (NOR) and Novel Object Location (NOL).

**Result:**

Our confocal imaging show a reduced density of the excitatory projections from LEC and MEC neurons in hippocampus compared to controls. Likewise, when evaluated the cognitive performance in NOR and NOL tasks, mice have a cognitive impairment performance in NOR and NOL.

**Conclusion:**

Our findings suggest that during an age period when Alzheimer’s disease phenotypes are already established, there is a markedly reduction in the connectivity between LEC, MEC and hippocampus with an episodic memory performance impaired. Current analysis to determine the precise period of the cortico‐hippocampal dysfunction appears during the disease progression are underway.

